# School performance after experiencing trauma: a longitudinal study of school functioning in survivors of the Utøya shootings in 2011

**DOI:** 10.3402/ejpt.v7.31359

**Published:** 2016-05-10

**Authors:** Ida Frugård Strøm, Jon-Håkon Schultz, Tore Wentzel-Larsen, Grete Dyb

**Affiliations:** 1 Norwegian Centre for Violence and Traumatic Stress Studies, Oslo, Norway; 2 Department of Education, University of Tromsø, The Arctic University of Norway, Tromsø, Norway; 3 Centre for Child and Adolescent Mental Health, Eastern and Southern Norway, Oslo, Norway; 4 Institute of Clinical Medicine, University of Oslo, Oslo, Norway

**Keywords:** Terror event, trauma, adolescents, school performance, registry grades, absenteeism, school support

## Abstract

**Background:**

The psychological impact on survivors of terrorism has been well documented. However, studies on adolescent survivors and the academic performance of high school students following a terrorist attack are lacking.

**Objective:**

This study investigated academic performance, absenteeism, and school support amongst survivors of a terrorist attack in Norway.

**Method:**

Data from a longitudinal interview study were linked to officially registered grades of students (*N*=64) who successfully completed their 3-year senior high school program. Statistical tests of mean differences and linear regression were used to compare the survivors’ registered grades with the national grade point average, before and after the event, as well as to assess absenteeism, self-reported grades and to test the association with school support.

**Results:**

The students’ grades were lower the year after the event than they had been the year before, and they were also lower than the national grade point average (*p*<0.001). However, their grades improved in the last year of high school, indicating possible recovery. Absence from school increased after the event, compared to the previous year. However, students reported high satisfaction with school support.

**Conclusion:**

The results indicate that academic functioning was reduced in the year after the traumatic event, but for students who successfully completed high school, the school situation improved 2 years after the event. The findings underscore the importance of keeping trauma-exposed students in school and providing support over time. A more defined educational approach to maintaining school attendance and educational measures which compensate for learning loss are needed in trauma-sensitive teaching.

**Highlights of the article:**

- School functioning among high school students following a terrorist attack.
- The findings showed a decline in school performance and increased absence after the event.
- For students who successfully completed high school, high satisfaction with school support was reported and an improvement in grades could be observed over time, indicating possible recovery.
- The findings underscore the importance of keeping trauma-exposed students in schools and providing support over time.

Several studies have focused on behavioral problems and psychological distress following a terrorist attack (Comer et al., 2010; Dyb, Jensen, Nygaard, et al., 2014; Galea et al., 2002; Moscardino, Scrimin, Capello, & Altoé, 2011; Nader, Pynoos, Fairbanks, & Frederick, 1990; Pynoos et al., 1987; Suomalainen, Haravuori, Berg, Kiviruusu, & Marttunen, 2011; Thoresen, Aakvaag, Wentzel-Larsen, Dyb, & Hjemdal, 2012). The negative impact of trauma on cognition is being increasingly recognized and the new symptom cluster “Negative alterations in cognitions and mood” was recently included in DSM-5 (American Psychiatric Association, 2013). However, few studies have focused on children and adolescent survivors and school functioning after experiencing a terrorist attack (Scrimin et al., 2006; Scrimin, Moscardino, Capello, & Axia, 2009).

On July 22, 2011, a car bomb exploded in the government quarter in Oslo, Norway, killing eight people and wounding several others. The perpetrator then drove to the small island of Utøya, where the youth organization of the Labor Party was hosting an annual summer camp, attended by 564, mostly young, people. The perpetrator gained access to the island by wearing a police uniform and pretending to be a police officer arriving to protect the youth. Upon reaching the island, he began a massacre which continued for more than 60 min, killing 68 persons, before the police took him into custody. In addition, many were injured and one more person died in hospital (Dyb, Jensen, Glad, et al., 2014).

This study will focus on the way in which this event affected survivors’ academic performance, absenteeism and response to school support. Adolescents’ school performance is of great importance for prospective higher education choices and consequently their long-term careers (Strøm, Thoresen, Wentzel-Larsen, & Dyb, 2013). Thus, if interrupted, it may have a negative impact on a person's life. A multitude of studies have focused on school functioning in the aftermath of trauma, including studies on children exposed to abuse (Leiter & Johnsen, 1997; Veltman & Browne, 2001), war (Elbert et al., 2009; Husain, Allwood, & Bell, 2008), disaster (Siriwardhana, Pannala, Siribaddana, Sumathipala, & Stewart, 2013; Weems et al., 2013), and death (Abdelnoor & Hollins, 2004; Berg, Rostila, Saarela, & Hjern, 2014; Park et al., 2014). Unfortunately, various methods and measures of school functioning have been applied and findings are not directly comparable. However, in a recent review on acute and chronic trauma events by Perfect et al. (2016), the main findings indicated that cognitive functioning, academic functioning, and teacher-reported social–emotional–behavioral functioning were affected by traumatic events. Majority of the trauma research on school functioning has focused on the consequences of chronic forms of trauma, wherein several studies have found an association between exposure to abuse and poor academic performance (Crozier & Barth, 2005; Hoffman-Plotkin & Twentyman, 1984; Kendall-Tackett & Eckenrode, 1996; Leiter & Johnsen, 1994; Perez & Widom, 1994; Slade & Wissow, 2007).

However, a terrorist attack is an acute single event, often characterized by severe injuries and deaths, which may produce different reactions and challenges for survivors. This distinction has not been clearly made in the current literature (Perfect et al., 2016). The Utøya event was unique in that all of the individuals involved were exposed to a high level of danger. The youths were isolated on an island and the only way to escape was by risking their lives swimming across the cold fjord. All of the adolescents heard gun shots and the majority of them heard or saw someone being injured or killed and were afraid that they were going to die themselves. Moreover, 74.5% reported that they lost someone close to them in the attack (Dyb, Jensen, Nygaard, et al., 2014). Thus, in this acute event, survivors were exposed to high levels of danger and loss. It was therefore expected that the event might impact their daily functioning and school performance.

The few studies conducted on students’ academic performance after a terrorist attack have mainly focused on specific cognitive functions, such as attention and memory (Scrimin et al., 2006, 2009), shifting, inhibition, and spatial working memory (Melinder, Augusti, Matre, & Endestad, 2015). These studies showed mixed results. Scrimin et al. (2006, 2009) conducted two studies on school-aged children who had survived the Beslan school terrorist attack and found that they had difficulties in sustaining attention and in short-term memory, as well as limited visual–spatial performance and lower grades compared to the non-exposed children (3 and 20 months after the attack). Melinder et al. (2015) examined long-term posttraumatic stress reactions and cognitive functions in 24 survivors of the Utøya shootings and compared the results to a control group. They found that one-third of the Utøya sample suffered from PTSD. However, the Utøya group did not differ significantly from the control group in cognitive functioning or school performance based on their final grades from high school.

These results are in alignment with studies on other acute traumatic events (war- and disaster-exposed children and witnessing death) and cognitive functioning which have also shown mixed results (Elbert et al., 2009; Hadi & Llabre, 1998; Park et al., 2014).

Studies of these types of traumatic events and academic achievement have also been mixed (Abdelnoor & Hollins, 2004; Berg et al., 2014; Elbert et al., 2009; Saigh et al., 2006; Weems et al., 2013). In a discotheque fire in Gothenburg, Sweden, 63 adolescents were killed and 213 physically injured. In a follow-up study of 275 victims 18 months after the event, the findings showed a severe impact on schoolwork. As many as 59% reported difficulties with schoolwork, poorer exam results, and lower grades (Broberg, Dyregrov, & Lilled, 2005). A limitation of this study was that many of the victims were immigrants with a history of trauma and stress. Thus, it is difficult to determine whether the effects were the result of previous traumatic experiences or the fire itself. Another study by Weems et al. (2013) showed no direct relationship between hurricane-exposed children and academic achievement but found an indirect relationship between PTSD and test anxiety. A study looking at the long-term outcomes of children who had experienced the Gulf crisis found a negative development in both educational and occupational outcomes as young adults (Hadi, Lai, & Llabre, 2014). Thus, further research is warranted to determine whether there is a direct linkage between exposure to a traumatic event and academic achievement, especially after a terrorist attack as research is sparse.

Adolescents spend much of their time in school, making it a crucial setting to study as the social relationships established there may protect against some of the negative outcomes associated with trauma. Studies on children exposed to disaster and community violence have shown association with lower school attendance, absenteeism (Mathews, Dempsey, & Overstreet, 2009; Siriwardhana et al., 2013), and dissatisfaction with school (Sims, Boasso, Burch, Naser, & Overstreet, 2015). However, research has shown that a positive school atmosphere is associated with school connectedness, higher academic achievement, and decreased high school dropout rates (Cohen, 2013; Haynes, Emmons, & Ben-Avie, 1997; Seilström & Bremberg, 2006). It is especially important to study the school setting when investigating the environment of individuals exposed to trauma as it may serve as a protective factor against some of its negative consequences, potentially affecting adolescent development in a positive way (Killen, 2009; Swenson & Chaffin, 2006).

Research on school support and its association with academic performance following trauma is limited. School support is here defined as sustainable efforts developed by the school to support trauma-exposed youth. A study by Yablon (2015) showed the importance of a positive school climate as a resilience factor for explaining PTSD and posttraumatic growth in high school students living in an armed conflict zone. However, several studies report that teachers are uncertain of how to support children who have been exposed to trauma and facilitate the learning process in these situations (Alisic, Boeije, Jongmans, & Kleber, 2012; Dyregrov, Dyregrov, & Idsoe, 2013; Papadatou, Metallinou, Hatzichristou, & Pavlidi, 2002). A review of 19 studies of school-based intervention programs for PTSD symptoms concluded that school-based interventions can be effective in helping children and adolescents following traumatic events (Rolfsnes & Idsoe, 2011). These interventions are often efforts from professionals external to the school system. However, there are a growing number of studies on creating trauma-informed schools with focus on school service delivery and a more comprehensive integration of trauma-informed approaches into the larger school context (Fu & Underwood, 2015; Green et al., 2015; Overstreet & Chafouleas, 2016). The Utøya survivors were mostly students in upper secondary school or higher education programs. The school semester started 4 weeks after the massacre, and the Ministry of Education instructed schools through two information letters to contact all students to plan their return to school and to tailor possible adaptations throughout the school year. The Norwegian Directorate of Education and Training also posted detailed information on its website about pupils’ rights to educational adaptations, including information from the Norwegian Education Act (2006) concerning permitted absence and alternative ways of assigning grades and completing classes in high school when students have high levels of absence. Teachers and school health workers were asked to be proactive and provide the survivors with close follow-up, supporting them to complete their school program.

This study will address some of the limitations in the field by not only looking at cognitive functioning in terms of academic achievement but also looking at absenteeism and the impact of school support on grades. The aims were to:

1. investigate the Utøya students’ academic performance, using objective measures of grades, from before and after the event, compared to the national grade point average,
2. examine the Utøya students’ absence from school before and after the event, and
3. study the level of school support, as perceived by students, and to what degree such support was associated with academic performance.

## Method

### Procedures

Of the 495 survivors of the terror attack, 490 (at least 13 years of age) were sent postal invitations 5 months after the event and were subsequently contacted by phone. Some survivors could not be reached by phone or declined to participate (*n*=165), while 325 (66.3%) were interviewed face to face. There were no significant differences in gender or age between participants and non-participants. Most of the semi-structured interviews (95.4%) were conducted by trained health personnel in November and December 2011. Survivors’ current needs for health services were assessed and interviewers provided help in contacting the appropriate resources. The study was based on written consent and was approved by the Regional Committee for Medical and Health Research Ethics in Norway (for details, see Dyb and Stene 2016).

The National Education Database (NUDB) provides information about citizens’ educational progress from junior high school until the completion of higher education. Information about the Utøya respondents’ grade point average and the national grade point average was submitted to us by the Norwegian Directorate for Education and Training. This allowed us to relate the questionnaire responses for each of the Utøya students to information about their academic performance, self-reported grades, absenteeism, and school support.

### Participants

The total Utøya study population included 325 persons, of which 268 reported that they were either full- or part-time students (Supplementary file, Fig. 1). Of these, 42 individuals did not have registered high school grades in the school years 2011–2014, possibly because they were university students. Among the remaining 226 student survivors registered in high school during 2011–2014, 20 individuals were excluded because they had missing consent (*N*=6), or had not accepted linkage of registry data (*N*=12). Of the sample who consented and had accepted linkage to registry data (*N*=208), the three largest age cohorts were chosen for the analyses: the 1993 cohort (*N*=50), the 1994 cohort (*N*=64), and the 1995 cohort (*N*=45). These cohorts were selected as they had measures of academic performance before and after the event. The remaining cohorts were excluded because they were small in size and only had measures before the event occurred (1988–1992 cohorts; *N*=35) or they only had measures from their first years of high school in 2012–2014 (1996–1997 cohorts, *N*=14).

**Fig. 1:**
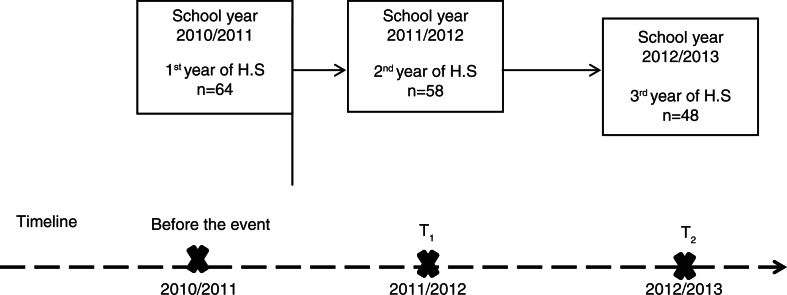
Timeline for data collection of the 1994 cohort.

The main focus of this article is on the 1994 cohort, which attended the first year of high school before the event, and the second and third year of high school after the event (Fig. 1). The measures were optimized by studying this group, as we had information on their academic performance before the event and at two time points after the event occurred. In order to organize the data, we only focused on the students who successfully completed high school within the ordinary time frame of 3 years.

### Measures

*Sociodemographic variables* included *age*, *gender*, and *ethnicity*. The latter was measured by asking if the respondent had parents who were born abroad. Non-Norwegian origin was defined as both parents having been born abroad.

#### Dependent variable

Academic performance was measured by using registry data for the students’ grade point average (ranging from 0 to 6), multiplied by 10 (ranging from 0 to 60). This score includes grades in all subjects as well as written and oral exams and is a standard method for listing grades in Norway. The national grade point average was calculated for each school year and grade by a statistician at the Norwegian Directorate for Education and Training.

#### Independent variables

*Subjective academic performance* was assessed by asking whether the respondents believed that their school performance had changed since the Utøya event, with the response options of (1) worse, (2) unchanged, or (3) better.

*School support* was assessed by three questions at T1, asking the respondents (1) whether the school had contacted them to facilitate their school start, (2) whether the school had done anything special to safeguard their teaching situation as a consequence of being at Utøya, or (3) whether they had a school staff member to talk to if necessary. The questions had a dichotomous response format (yes or no). At T2, school support was assessed by two questions asking respondents (1) whether they had received extra support in the previous school year with a dichotomous response format (yes or no) and (2) how satisfied they were with the support/facilitation provided by the school in the previous year. The possible responses for the latter question ranged from “not at all” to “to a very high degree.”

#### Statistical methods

A one-sample *t*-test was conducted to compare the Utøya students’ academic performance with the national grade point average. Furthermore, mixed-effects analyses were used to compare the Utøya students’ grades, as well as hours and days of absence before and after the event. To test the relationship between self-reported grades and registry grades, a one-way ANOVA was applied. Finally, linear regression analysis was used to test the relationship between school support and academic performance.

IBM Statistics 22 was used for all analyses with the exception of the mixed-effects analyses, which used the R package nlme.

## Results

### The 1994 cohort

The total study sample consisted of 64 students (57.8% girls), ranging from 16 to 18 years (first–third years of high school (HS) and the majority of the students had parents born in Norway (95.2%, *n*=60). Of the 64 students who had completed the first grade of high school before the event, 6 did not start second grade and 10 of the second graders did not start the final year of high school. This constitutes a total of 16 students who did not successfully complete high school after the Utøya event (26.6%). However, no students dropped out of school once they had started a semester. The grades ranged from 0 to 56, of which the mean grade in the first year of high school (before the event) was 38.53, the second year (after the event) was 34.64, and in the final year (after the event) it was 38.96.

### The Utøya students’ academic performance before and after the event

Considering only the Utøya cohort, the mixed-effects analyses showed that, compared to their academic performance before the event, the students’ grades in the second year of high school had dropped significantly by 5.51 (*p*<0.001) grade points. By the last year of high school, their grades were still lower than before the event (−3.07, *p*=0.004) but somewhat better than the year before (Table 1).

**Table 1:** Mixed-effects analyses comparing Utøya students’ academic performance before and after the Utøya shootings

	Mean value	95% CI for mean difference	*p*
Comparing second year HS with first year HS	−5.51	−8.23, −2.79	0.001
Comparing third year HS with first year HS	−3.07	−6.0, −0.15	0.039

HS, high school.

### Comparing the Utøya students’ academic performance with the national grade point average

In the year before the Utøya shootings, the Utøya cohort's academic performance was comparable to the national grade point average (Table 2). However, the year after the event, their grades dropped by an average of 4.3 points compared to the national score. This trend turned around in the last year of high school (2 years after the event) as their academic performance returned to a level similar to the national grade point average.

**Table 2:** One-sample *t*-test analyses of the difference between Utøya students’ academic performance and national grade point average

	*M*	SD	*n*	95% CI for mean difference	*p*
Before the event: first year of HS	0.13	10.71	64	−2.54, 2.81	0.920
After the event: second year of HS	−4.26	12.22	58	−7.47, −1.05	0.010
After the event: third year of HS	−0.04	11.10	47	−3.30, 3.22	0.980

HS, high school.

### School absence before and after the terrorist attack

The average hours of absence before the event was 16.1 (SD=16.4, *N*=64), while the mean value for days of absence was 6.0 (SD=5.1, *N*=64). Of the enrolled students, a large increase in the absence for both days and hours could be observed after the event took place compared to the year before the event (Table 3). In each school year, up to 10 days of absence is allowed, for example for medical appointments. If properly documented, these can be subtracted from the reported total sum.

**Table 3:** Mixed-effects analyses comparing the Utøya students’ days and hours of absence before and after the Utøya shootings

		Mean value	95% CI for mean difference	*p*
Comparing second year HS (after the event) with first year HS (before the event)	Days of absence	12.19	7.50, 16.89	0.001
	Hours of absence	37.00	25.89, 48.03	0.001
Comparing third year HS (after the event) with the first year HS (before the event)	Days of absence	8.32	3.24, 13.40	0.001
	Hours of absence	33.00	21.01, 44.92	0.001

HS, high school.

### Comparing the self-reported grades with the registry grades

The year after the event (school year 2011/2012), 75% (*N*=42) of the Utøya respondents reported that they felt that their school performance had worsened compared to before the event, 16.1% (*N*=9) reported that they were unchanged and a small percentage (8.9%, *N*=5) felt that they had performed better than before the event. The one-way ANOVA test was not significant but showed differences between registry and self-reported grades (Table 4).

**Table 4:** ANOVA analyses comparing the subjective answers on school support received after the terrorist attack with registry grades

	Second year of HS grades compared to before the event		Third year of HS grades compared to before the event	
		
	*M*	SD	*M*	SD
Worse	−8.21	10.71	−3.58	8.74
Unchanged	−0.911	12.22	−1.13	7.11
Better	−3.52	11.12	1.13	3.75

School year 2011/2012: *F*(56)=2.38, *p*=0.102. School year 2012/2013: *F*(38)=1.07, *p*=0.354.

### School support and academic performance

Finally, we wanted to investigate whether the students’ experience of school support was related to their academic performance. The students expressed high satisfaction with the school support that they had received after the terrorist attack. The year after the event, 84.5% (*n*=49) reported that the school had contacted them to facilitate their school start, 87.7% (*N*=50) reported that the school had done something special to safeguard their teaching situation as a consequence of being at Utøya, and 89.5% (*n*=51) reported that they had a school staff member to talk to if necessary. During the last year of high school, 84.2% (*n*=32) reported that they received extra support the previous school year and 63.9% (*n*=23) said that they were highly satisfied with the support/facilitation provided by the school the previous year. However, we did not find any significant relationship between school support and grades after the event.

### Overview of the 1993 and 1995 cohorts

The trend seen in the 1994 cohort could not be observed in the 1993 and 1995 cohorts. The 1993 cohort was academically weaker than the national grade point average (*p*=0.033) before the event (Supplementary file, Table 1) and was still performing below the national average after the event, but somewhat better than the year before, although not significantly. Studying only the Utøya cohort, there was no significant change in grades before and after the event.

The 1995 cohort was in the tenth grade before the event and had a higher average score than the national grade point average (*p*=0.005) (Supplementary file, Table 2). However, after the event, their high school grades were comparable to the national grade point average, with the exception of the second grade where the grades dropped somewhat, but no differences were significant (Supplementary file, Table 3). This cohort's grades could not be compared as they had not started high school in 2010/2011 and academic performance in tenth grade is not comparable to high school grades.

## Discussion

High school performance may determine educational career. Thus, if interrupted or weakened it may have a negative impact on a person's life. As expected, our results showed that trauma-exposed students performed worse academically the year after experiencing the terrorist attack. They also had lower grades than the national grade point average. These results can be seen in light of what the students experienced. The majority of the students felt that their lives were in danger, many witnessed people dying, and as many as 75% of the respondents reported that they had lost someone close to them. It is natural to anticipate that this would impact their school functioning as they began school only 4 weeks after the event. It can be assumed that their school performance may have been affected by posttraumatic stress reactions, grief, lack of sleep, and pain after experiencing the traumatic event (Dyb, Jensen, Nygaard, et al., 2014).

These results are in alignment with research on similar forms of traumatic events such as bereavement, exposure to war and other natural disasters, which have been found to associate with poorer academic achievement (Abdelnoor & Hollins, 2004; Berg et al., 2014; Elbert et al., 2009; Hadi et al., 2014; Perfect et al., 2016). For example, Broberg et al. (2005) reported students dropping out and having difficulty with school performance after experiencing a discotheque fire. Similarly, Scrimin et al. (2009) found that trauma-exposed students’ school grades following a terrorist attack were significantly lower compared to the controls. They also studied physical proximity to the event, personal loss, and multiple exposures to traumatogenic elements to find out whether the students who had been directly exposed would perform worse than the indirectly exposed students, but no significant differences were found between the groups. However, they did find poorer memory function among the directly exposed students. This is relevant to our study sample as all the Utøya participants were directly exposed to the event and experienced several traumatogenic elements. Perfect et al. (2016) concluded in their review that youth with more severe exposure to traumatic events were at increased risk for poorer cognitive functioning, academic difficulties, and social–emotional–behavioral problems.

Considering the impact on the long-term functioning of children's developing brains, Scrimin et al. (2009) argue that a single terrorism-related traumatic event may be similar to other chronic forms of trauma and their association with cognitive functioning. Moreover, because a terror event can happen any time at any place, the threat of terrorism is enduring and ever-present. In our study, the students who successfully completed high school performed somewhat better in school as time passed, possibly indicating that they had the support and the capacity to perform better. Which factors could explain this change? Previous studies have shown that the victims of the Utøya shootings have been struggling with poor mental health since the event (Dyb, Jensen, Nygaard, et al., 2014). So how were they still managing to do relatively well in school 2 years after the event? One possible explanation is school support. A majority of the students reported that the school had contacted them before school began and had facilitated the start of the semester and teaching situation as well as providing them with someone to talk to if necessary the year after the event. During the last year of high school, the majority of the students reported that they had received extra support and about two-thirds of the respondents reported that they were highly satisfied with the support given by the school the previous year. Thus, the students expressed a high level of satisfaction with the received school support. However, these items primarily reflect a psychosocial, rather than an educational adaptation which may explain why there was no significant association between school support and grades.

Another measure that may give an indication of students’ adjustment to school is the level of absenteeism. Our results showed a large increase in both hours and days of absence compared to the year before the event, indicating that the students may have struggled in school. Dyregrov, Dyregrov, Endsjø, and Idsoe (2015) found similar results in a study of teachers’ perception of bereaved children's academic performance in which the teachers reported increased absence among high school students. However, the level of absence in our study decreased somewhat in the second year after the event, and no students dropped out of school once they were enrolled in a semester.

There is a lack of research on the school's role after trauma (Broberg et al., 2005). Some research has focused on the role of teachers (Alisic et al., 2012; Dyregrov et al., 2013, 2015; Papadatou et al., 2002) and some on school interventions (Murtonen, Suomalainen, Haravuori, & Marttunen, 2012; Rolfsnes & Idsoe, 2011), but few studies, if any, have looked at the school's facilitation after a terrorist attack and how it has affected the students’ academic achievement. This study emphasizes the importance of investigating the students’ school performance and the school's role following a traumatic event. More studies are needed to investigate the school's role and its effect on grades and to consider measures for keeping students enrolled in school.

Other possible explanations for the improvement in grades 2 years after the event and the low dropout rate once enrolled, may be, as mentioned by Scrimin et al. (2009), that the schools were more lenient toward the victimized students, possibly resulting in better grades. The Norwegian Ministry of Education's instructions to the schools on providing support and making possible adaptations for the affected students, along with the Norwegian Directorate of Education and Training's information about the students’ right to adaptation, might have resulted in the students managing well in school and maintaining their grades over time. The flexibility provided by the Norwegian Education Act (2006) concerning legal absence and alternative ways of assigning grades, when students have high levels of absence, may also have influenced the outcome, although this was not tested in this study. This also applies to the other two cohorts, which showed no significant change in grades after the event.

## Conclusion

The results of this study showed that the Utøya students’ academic performance was worse the year after the event than it had been the year before and also that their grades were lower than the national grade point average. However, their grades improved in the last year of high school. The same pattern could be observed in the number of registered absences, in which there was a large increase in both days and hours of absence the year after the event but it decreased somewhat during the last year of high school. Finally, the students reported high satisfaction with the school support they received, although this was not significantly related to grades. In conclusion, it is unclear what caused the students to perform better over time. School attendance and school support provided to the students who successfully completed high school on time may have been central in utilizing the students’ capacity for recovery. However, these results need to be interpreted with caution considering that we only included the students who successfully completed high school within 3 years.

### Strengths and limitations

The design of the Utøya study (Dyb, Jensen, Glad, et al., 2014) is strengthened by the relatively high response rate and low levels of missing data. Furthermore, the study was performed shortly after the disaster, thereby adding to the limited knowledge of victimized students’ school performance in the acute aftermath (Dyb, Jensen, Nygaard, et al., 2014). Moreover, it included objective measures of grades before and after the event which we were able to compare to the national grade point average. In addition, it had a broad perspective on school functioning covering many aspects of the school situation, rather than focusing only on cognitive functioning as previous studies have done.

The following limitations are relevant when interpreting the results of this study. We only followed students who successfully completed high school within 3 years. It would be interesting to further investigate those students who dropped out of high school after the event and the associated factors explaining this outcome. There was no comparison group for the level of absence. Thus, we cannot tell whether the level of absence after the event was above or below the average national mean. Furthermore, the distribution of absence among the Utøya students was skewed, limiting the results of the mixed effect analyses.

However, despite these limitations, this study is unique in that it addresses several of the current limitations in the field and it is the first study to examine students’ school performance following a terrorist attack, using objective measure of grades.

### Implications

Findings in this study contribute trauma-specific knowledge to the fields of education, special needs education, and educational psychology, where there is currently scarce research on how to facilitate the learning situation for pupils suffering from traumatic stress. Our findings underscore the importance of keeping trauma-exposed students in school and providing support over time. Maintaining school attendance and school support appear to be of importance in order to utilize students’ capacity for recovery over time. More research is needed to investigate the specific efforts conducted by the school to maintain students’ academic performance and low dropout rate once enrolled in the semester. With regard to practice, educational staff need to be aware of students’ reactions after a traumatic event and the possible effect on their school performance. In particular, we need a better understanding of what school support entails regarding practical educational adaptations targeting reduced learning capacity due to trauma. A more defined educational approach to maintaining school attendance and educational measures, which compensate for learning loss, should be natural components in trauma-sensitive teaching.

## Supplementary Material

School performance after experiencing trauma: a longitudinal study of school functioning in survivors of the Utøya shootings in 2011

School performance after experiencing trauma: a longitudinal study of school functioning in survivors of the Utøya shootings in 2011

School performance after experiencing trauma: a longitudinal study of school functioning in survivors of the Utøya shootings in 2011
