# Supplementary material for: School performance after experiencing trauma: a longitudinal study of school functioning in survivors of the Utøya shootings in 2011
Source: Eur J Psychotraumatol. 2016 May 10;7:10.3402/ejpt.v7.31359. doi: 10.3402/ejpt.v7.31359 (PMC4864847; doi:10.3402/ejpt.v7.31359)
Supplement: School performance after experiencing trauma: a longitudinal study of school functioning in survivors of the Utøya shootings in 2011 [file EJPT-7-31359-s001.pdf]

School performance after experiencing trauma: A longitudinal study of school functioning in survivors of the Utøya shootings in 2011

Ida Frugård Strøm, Jon-Håkon Schultz, Jon-Håkon Schultz, Tore Wentzel-Larsen, Tore Wentzel-Larsen, Grete Dyb, Grete Dyb

Bakgrunn: Psykologiske konsekvenser blant overlevende som har opplevd terrorisme er godt dokumentert i litteraturen. Få studier har fokusert på ungdom som har overlevd et terrorangrep og hvordan skoleprestasjonene deres har blitt påvirket som en følge av dette.
Målsetting: Denne studien undersøker skoleprestasjoner, fravær og skolestøtte hos ungdommer som overlevde Utøya hendelsen.
Metode: Data fra en longitudinell intervjustudie ble koblet til offisielt registrerte karakterer for tre studentkohorter som suksessfullt gjennomførte videregående skole (N=159). I denne artikkelen har vi valgt å fokusere på en av studentkohortene, 1994 kohorten(N=64), da vi har et mål fra videregående skole før hendelsen og to mål etter hendelsen. Statistiske tester av gjennomsnittsforskjeller og lineær regresjon ble gjennomført for å sammenlikne registerkarakterene til de overlevende med det nasjonale gjennomsnittet før og etter hendelsen samt for å undersøke fravær, selvrapporterte karakterer og sammenhengen med skolestøtte.
Resultater: Karakterene til studentene (1994 kohort) som overlevde Utøya var lavere året etter hendelsen sammenliknet med året før hendelsen, og de var også lavere enn det nasjonale gjennomsnittet ($p<0.001$). Karakterene forbedret seg det siste året på videregående, to år etter hendelsen, noe som indikerer en mulig forbedring. Det var ingen forskjell i karakterer før og etter hendelsen i de to andre studentkohortene. Fravær fra skolen økte etter hendelsen. Elevene var i stor grad fornøyd med skolestøtten de mottok.
Konklusjon: Resultatene indikerer at skoleprestasjonene ble dårligere (1994 kohort) etter å ha opplevd et terrorangrep, men for studentene som suksessfullt gjennomførte videregående, så forbedret skolesituasjonen seg to år etter hendelsen. Disse funnene legger vekt på hvor viktig det er å holde traumeeksponerte elever i skolen, samt å bidra med støtte over tid. Det er behov for en mer definert tilnærming til hvordan man kan vedlikeholde skolegang og karakterer hos traumeeksponerte barn og unge som følge av tapt undervisning.

Citation: European Journal of Psychotraumatology 2016, **7**: 31359 - http://dx.doi.org/10.3402/ejpt.v7.31359
