# Supplementary material for: School performance after experiencing trauma: a longitudinal study of school functioning in survivors of the Utøya shootings in 2011
Source: Eur J Psychotraumatol. 2016 May 10;7:10.3402/ejpt.v7.31359. doi: 10.3402/ejpt.v7.31359 (PMC4864847; doi:10.3402/ejpt.v7.31359)
Supplement: School performance after experiencing trauma: a longitudinal study of school functioning in survivors of the Utøya shootings in 2011 [file EJPT-7-31359-s002.pdf]

Radzenie sobie w szkole po traumie strzelaniny na wyspie Utøya w 2011 r. wśród osób ocalałych - badanie podłużne.

Ida Frugård Strøm, Jon-Håkon Schultz, Jon-Håkon Schultz, Tore Wentzel-Larsen, Tore Wentzel-Larsen, Grete Dyb, Grete Dyb

Wprowadzenie: Psychologiczne skutki ataków terrorystycznych są dokładnie udokumentowane. Niewiele jednak wiadomo na temat skutków ataku terrorystycznego na radzenie sobie w szkole przez osoby, które taki atak przeżyły.
Cel: Celem niniejszego badania była ocena radzenia sobie w szkole, przejawiającego się w wynikach szkolnych, liczby nieobecności oraz korzystania ze wsparcia społecznego w szkole wśród osób ocalałych po traumie strzelaniny na wyspie Utøya w Norwegii.
Metoda: W badanu podłużnym uczestniczyła próba 64 uczniów trzeciego roku szkoły sredniej, którzy przeżyli tego rodzaju traumę.
Wyniki: Wykazano znaczace obniżenie ocen szkolnych ($p<0.001$) rok po tragedii w porównaniu do funkcjonowania szkolnego uczniów rok przed tragedią. Zauważono tez istotny wzrost nieobecnosci szkolnych. Jednakże odnotowano wzrost satysfkacji z korzystania z wsparcia społecznego w szkole.
Konkluzje: Wyniki tych badań wskazują na znaczące obnizenie ocen szkolnych oraz wzrost nieobecności wśród uczniów, którzy przezyli nieniejsza tragedie. Jednakże odnotowano też wzrost satysfakcji ze wsparcia społecznego otrzymywanego w szkole, co może nastrajac optymistycznie na temat radzenia sobie w szkole po traumie ataku terrorystycznego.

Słowa kluczowe: atak terrorystyczny, trauma, nastolatek, radzenie sobie w szkole, oceny szkolne, nieobecność, wsparcie społeczne w szkole.

Name of translator: Marcin Rzeszutek, University of Finance and Management in Warsaw, Poland

Citation: European Journal of Psychotraumatology 2016, **7**: 31359 - http://dx.doi.org/10.3402/ejpt.v7.31359
