# Supplementary material for: School performance after experiencing trauma: a longitudinal study of school functioning in survivors of the Utøya shootings in 2011
Source: Eur J Psychotraumatol. 2016 May 10;7:10.3402/ejpt.v7.31359. doi: 10.3402/ejpt.v7.31359 (PMC4864847; doi:10.3402/ejpt.v7.31359)
Supplement: School performance after experiencing trauma: a longitudinal study of school functioning in survivors of the Utøya shootings in 2011 [file EJPT-7-31359-s003.pdf]

Title: Школьная успеваемость после травмы: лонгитюдное исследование школьной деятельности учащихся уцелевших в обстреле на острове Утойя в 2011 году

Ida Frugård Strøm, Jon-Håkon Schultz, Jon-Håkon Schultz, Tore Wentzel-Larsen, Tore Wentzel-Larsen, Grete Dyb, Grete Dyb
Ида Фругард Штром, Джон-Хакон Шульц, Торе Вентзел-Ларсен, Грете Диб

Abstract
Исходная информация: Психологические последствия терроризма - хорошо исследованная область. В тоже самое время, малоизучены в этом плане подростки и школьная успеваемость старшеклассников, подвергшихся террористической атаке.
Цель: Данное исследование изучило академическую успеваемость, пропуски школы и школьную поддержку подростков уцелевших в теракте в Норвегии.
Метод: Данные, полученные путем лонгитюдных интервью, были соотнесены с официальными данными по успеваемости студентов (N= 64), которые успешно закончили учебу в трехлетней программе для старших классов. Статистические тесты минимальных различий и линеарной регрессии были применены для сравнения официальных школьных отметок уцелевших подростков со средними аналогичными показателями национального уровня до и после инцидента, а также, для оценки пропусков школы, самоотчетов по поводу оценок и выявления связи со школьной поддержкой.
Результаты: Отметки учеников в течении года после инцидента были ниже по сравнению с их величиной за год до инцидента; также, они были ниже среднего национального показателя ($p<0.001$). В тоже самое время, отметки уцелевших подростков улучшились в завершающий учебу в школе год, что может отражать возможное выздоровление. Пропуски школы участились после инцидента, по сравнению с предыдущим годом. В тоже самое время, среди исследованных учеников выявился высокий уровень удовлетворенности школьной поддержкой.
Выводы: Результаты исследования доказывают, что академическая деятельность уцелевших подростков понизилась в последующий за инцидентом год, но для тех учащихся кто успешно окончил школу, ситуация улучшилась через два года после инцидента. Результаты исследования подтвердили важность того, что бы учащиеся перенесшие травматический опыт находились в школьной среде, которая обеспечит им долгосрочную поддержку. Необходим более определенный образовательный подход для обеспечения посещаемости школы учащимися которые прошли через травматический опыт; также, подтвердилась необходимость чувствительных к травме образовательных методов для компенсации вызванных травматическим опытом затруднений в обучении.

Keywords: террористический инцидент, травма, подростки, школьная успеваемость, школьные оценки, пропуски школы, школьная поддержка

Name of translator: Jana Darejan Javakhishvili
Джана Дареджан Джавахишвили

Citation: European Journal of Psychotraumatology 2016, **7**: 31359 - http://dx.doi.org/10.3402/ejpt.v7.31359
